# Pharmacologic Modulation of Serine/Threonine Phosphorylation Highly Sensitizes PHEO in a MPC Cell and Mouse Model to Conventional Chemotherapy

**DOI:** 10.1371/journal.pone.0014678

**Published:** 2011-02-14

**Authors:** Lucia Martiniova, Jie Lu, Jeffrey Chiang, Marcelino Bernardo, Russell Lonser, Zhengping Zhuang, Karel Pacak

**Affiliations:** 1 Reproductive and Adult Endocrinology Program, Eunice Kennedy Shriver, National Institutes of Child Health and Human Development, National Institutes of Health, Bethesda, Maryland, United States of America; 2 Surgical Neurology Branch, National Institute of Neurological Disorders and Stroke, National Institutes of Health, Bethesda, Maryland, United States of America; 3 Experimental Immunology Branch, National Cancer Institute, National Institutes of Health, Bethesda, Maryland, United States of America; 4 Molecular Imaging Program, National Cancer Institute, National Institutes of Health, Bethesda, Maryland, United States of America; 5 Laboratory Animal Sciences Program, SAIC-Frederick, NCI-Frederick, Frederick, Maryland, United States of America; 6 Institute of Experimental Endocrinology, Slovak Academy of Sciences, Bratislava, Slovakia; Bauer Research Foundation, United States of America

## Abstract

**Background:**

The failure of cytotoxic cancer regimens to cure the most drug-resistant, well-differentiated solid tumors has been attributed to the heterogeneity of cell types that differ in their capacities for growth, differentiation, and metastases. We investigated the effect of LB1, a small molecule inhibitor of serine/threonine protein phosphatase 2A (PP2A), on its ability to inhibit a low growth fraction and highly drug-resistant solid neuroendocrine tumor, such as metastatic pheochromocytoma (PHEO). Subsequently, we evaluated the increased efficacy of chemotherapy combined with LB1.

**Methodology/Principal Findings:**

The effect of LB1 and temozolomide (TMZ), a standard chemotherapeutic agent that alone only transiently suppressed the growth and regression of metastatic PHEO, was evaluated *in vitro* on a single PHEO cell line and *in vivo* on mouse model of metastatic PHEO. In the present study, we show that metastatic PHEO, for which there is currently no cure, can be eliminated by combining LB1, thereby inhibiting PP2A, with TMZ. This new treatment approach resulted in long term, disease-free survival of up to 40% of animals bearing multiple intrahepatic metastases, a disease state that the majority of patients die from. Inhibition of PP2A was associated with prevention of G1/S phase arrest by p53 and of mitotic arrest mediated by polo-like kinase 1 (Plk-1).

**Conclusions/Significance:**

The elimination of DNA damage-induced defense mechanisms, through transient pharmacologic inhibition of PP2A, is proposed as a new approach for enhancing the efficacy of non-specific cancer chemotherapy regimens against a broad spectrum of low growth fraction tumors very commonly resistant to cytotoxic drugs.

## Introduction

Cancer therapy has been most successful against aggressive tumors characterized by a high population of cells in active cell growth (high-growth fraction), although acquired impairment of DNA-damage repair mechanisms may underlie the chemotherapy sensitivity of the most curable cancers [1]. Nevertheless, aggressive chemotherapy, combined at times with radiation, often cures several types of rapidly growing poorly differentiated cancers including leukemias, lymphomas, testicular cancers, and gestational choriocarcinomas, even though they are disseminated at diagnosis. This is not the case for the most common and more slowly growing cancers of the prostate, breast, lung, colon, and ovary, for which a cure is generally not achievable if the tumor cannot be eliminated completely by surgery/radiation. Whether these cancers have subsets of intrinsically resistant high-growth fraction cells or whether they are resistant to cytotoxic therapy simply because they are not in active cell division is not certain [2]. The distribution of cells in a given cancer at different phases of the cell cycle may be an important factor in determining the efficacy of cytotoxic treatment [3], [4]. Attempts to overcome cell-cycle dependent resistance have included administration of drugs to preferentially disturb the regulation of the cell-cycle and DNA-repair in cancers compared to the cells of normal tissues [5], [6], [7]. Regardless of the mechanisms of resistance, most solid malignant tumors have a cell population, which survives the most aggressive combinations of highly cytotoxic drugs given on a variety of schedules.

In the present study, we wanted to determine whether inhibition of PP2A also potentiates the effectiveness of temozolomide (TMZ) against well-differentiated low-growth fraction solid tumors, such as pheochromocytoma (PHEO). PHEOs arise from chromaffin cells of the adrenal gland. Comparable tumors arising from extraadrenal chromaffin cells are termed as paragangliomas (PGLs). Both tumors are characterized by the synthesis, storage and release of catecholamines [8]. Most PHEOs and PGLs are sporadic but about 25% are associated with familial disorders including multiple endocrine neoplasia type 2, neurofibromatosis type 1, von Hippel-Lindau syndrome, and syndromes associated with mutations of genes encoding subunits of the succinate dehydrogenase complex [9], [10], [11]. Most of these tumors are not malignant; however, about 40% of patients presenting with metastatic disease harbor an underlying mutation, most notably in the gene for subunit B of the succinate dehydrogenase complex [12], [13]. Because of their high degree of differentiation, malignant PHEOs and PGLs are diagnosed primarily by demonstrating the presence of aggregates of chromaffin cells in sites where chromaffin cells are not normally present and from specific biochemical abnormalities [14]. Although metastatic PHEOs and PGLs are generally slow growing, the prognosis of patients with disseminated disease is poor, with a 5-year survival rate less than 50%. This is due in large part to the fact that, currently, there is no effective chemotherapeutic regimen [15]. A long-term follow-up study conducted on 18 patients reported a complete response rate of 11% and a partial response rate of 44% of metastatic PHEO and PGL patients after cyclophosphamide, vincristine, and dacarbazine (CVD) treatment [16]. Moreover, recent studies demonstrated that the survival rate between patients treated with CVD chemotherapy and those without treatment did not differ [16], [17].

TMZ has been reported to have some therapeutic benefit in the treatment of metastatic neuroendocrine carcinomas, including malignant melanomas and PHEOs [18], [19], [20]. Because of the marked ability of LB-1.2 to sensitize xenografts of glioblastoma multiforme and neuroblastoma to TMZ, cancers also minimally inhibited by TMZ, [21] we studied the anti-tumor activity of LB1 (LB-1, LB-100), a water-soluble homolog of LB1.2 in combination with TMZ; each compound was also studied individually. All experiments were conducted against mouse PHEO cells (MPC cell line) *in vitro* and against a metastatic model *in vivo*. At present, the MPC cell line most closely resembles well-differentiated neoplastic human chromaffin cells including the expression of tyrosine hydroxylase and phenylethanolamine-N-methyltransferase [22], unique markers of catecholamine synthesis.

## Results

### In vitro and in vivo anti-tumor activity of LB1 and TMZ and their combination

**Figure 1:**
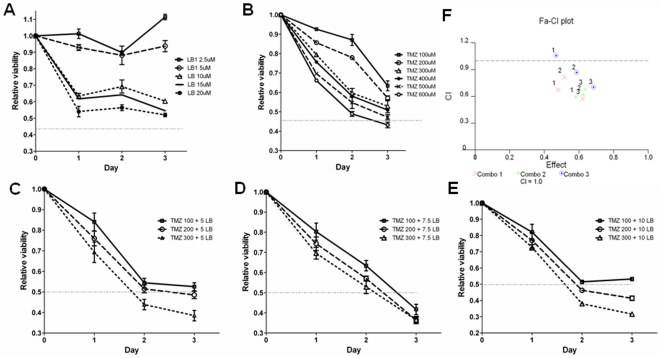
*In vitro* anti- proliferative activity of LB1 and TMZ and their combination. Inhibition of growth of MPC cells in culture: (A and B) Exposure for 3 days to increasing concentrations of LB1 or TMZ. (C–E) Exposure to increasing concentrations of LB1 plus TMZ. (F) Synergy analysis was done based on data from (A–E) using CalsuSyn software. CI values: C = 1 as additivity; C<1 as synergy; C>1 as antagonism. Combo 1 presents combination of 5 µM of LB1 and 100, 200, 300 µM of TMZ; Combo 2 presents combination of 7.5 µM of LB1 and 100, 200, 300 µM of TMZ; and Combo 3 10 µM of LB1 and 100, 200, 300 µM of TMZ.

*In vitro*, both LB1 and TMZ, at concentrations up to 20 µM and 600 µM, respectively, showed only modestly inhibited MPC cell proliferation. Even at maximum concentration tested, LB1 drug did not achieve over 50% inhibition and TMZ only with 600 µM concentration achieved over 50% inhibition (Figure 1A–1B). Both drugs showed slightly greater growth inhibition at concentrations of TMZ between 100 and 300 µM and combined with LB1 at concentrations between 5 and 10 µM (Figure 1C–1E). The combination index (CI) was used to confirm and quantify the synergy observed with LB1 and TMZ. CI values were <0.9 at almost all doses (range from 5–10 µM of LB1 and 100–300 µM of TMZ; Figure 1F), indicating that synergy occurred between LB1 and TMZ. There was no interference of drugs and XTT agent kit observed at the absence of cells.

**Figure 2:**
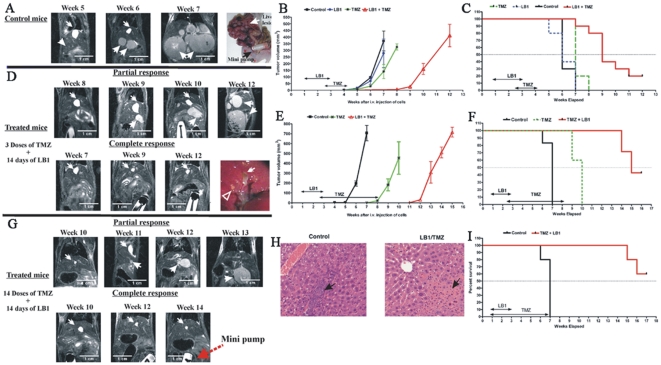
*In vivo* anti-tumor activity of LB1 and TMZ and histological examination. Effects of treatment upon growth and molecular changes in hepatic tumors: (A) MRI images of untreated mice at weeks 5, 6, and 7 following intravenous injection of MPC cells and a photomicrograph showing the growth hepatic lesions and Alzet minipump. Barbed arrow indicates the gall bladder. Plain arrows indicate the same tumor nodules over time. (B) Inhibition of total hepatic tumor volume by LB1 alone at 1.5 mg/kg daily for 14 days by *c.i.* administered at 5^th^ day after MPC cells injection; TMZ alone at 80 mg/kg for 3 doses administered at 15^th^ day after MPC cells injection; and, combination of both drugs. (C) Survival curve combining the data from the study depicted in (B) a total of 10 control animals, 10 animals with combination treatment LB1 and TMZ, 5 animals with TMZ alone, and 5 animals with LB1 alone. Kaplan-Meier analysis revealed that survival following LB1 plus TMZ was significantly greater than with LB1 alone and TMZ alone (log rank, *P*<0.0001). (D) MRI images of mice, treated with the combination of LB1 by *c.i*. for 14 days and 3 doses of TMZ, at week 7, 9, and 12. Partial response of treatment is presented with delayed appearance of hepatic tumors compared to untreated group. Complete response presents absence of hepatic tumors after treatment. A photomicrograph of the liver of one treated animal at week 12, showing the absence of gross tumor, and the presence of fibrous scar tissue (arrows). (E) Inhibition of estimated total hepatic tumor volume by combination treatment of LB1 and TMZ. LB1 at 1.5 mg/kg daily for 14 days by *c.i*. administered at 5^th^ day after MPC cells injection and TMZ at 80 mg/kg every 3 days for 14 doses beginning on 15^th^ day after MPC cells injection (with combination or alone). (F) Survival curve combining the data described in E. Total of 12 control animals, 5 animals for TMZ and 7 animals for combination treatment LB1 and TMZ. Survival of animals with combined treatment were significantly greater compared to controls (log rank, *P*<0.0001). (G) Serial MRI images of mice, treated with the combination of LB1 and 14 doses TMZ with partial and complete responses. (H) Histologic features of liver PHEO at week 12 stained with H&E receiving no treatment or LB1 by *c.i.* and three doses of TMZ as described in D. Untreated animals showed intrahepatic deposits of cancer cells whereas the liver of an animal receiving both drugs that had no gross evidence of tumor revealed normal parenchyma and fibrous tissue, believed to be scarring at former sites of tumor masses. (I) Survival curves of animals treated with LB1 and 14 doses of TMZ when administration started at the same time, on day 5 after MPC cells injection. (n = 5 treated animals with LB1 plus TMZ, n = 5 for controls; log rank, *P* = 0.0035).

We then tested drug efficacy against metastatic PHEO *in vivo.* Following intravenous tail vein injection, MPC cells formed multiple intrahepatic masses, detectable by magnetic resonance imaging (MRI) starting from the 4^th^ week post injection. Untreated, numerous hepatic tumor nodules reach total volumes, estimated from the MRI images, of 700±100 mm^3^ by week 7, requiring sacrifice of the animals (Figure 2A–2B). Control animals (untreated animals) received vehicle (phosphate buffered saline-PBS) alone by continuous infusion (*c.i*.) for 14 days via an Alzet capsule implanted intraperitoneally (*i.p*.) on the 5^th^ day after tumor cell injection. LB1 was given by *c.i*. at 1.5 mg/kg per day for 14 days starting on the 5^th^ day after tumor cell injection. TMZ was administered by gavage alone or with LB1 at 80 mg/kg every 3 days on 3 different schedules: 3 doses beginning 10 days from the start of LB1; 14 doses beginning 10 days from the start of LB1; and 14 doses beginning from the start of LB1.

LB1 alone (n = 5) had no inhibitory effect as estimated from MRI images. In contrary, liver tumors were growing more rapidly after 14-days of LB1 treatment, even compared to control group (Figure 2B,C, blue line), demonstrated by weekly MRI and volume measurements. Three doses of TMZ every 3 days alone slowed tumor growth slightly in all animals (n = 5; Figure 2B and C, green line), whereas combination of both drugs markedly reduced the rate of increase in hepatic tumor volume in all animals (n = 10 treated animals) compared to tumor volume rate in control animals (n = 10; Figure 2B and C, red line). Growth of tumors in animals given TMZ plus LB1 was delayed to week 9 (Figure 2B), with 2 of 10 animals having no apparent tumor at 12 weeks by serial MRIs (Figure 2D). After sacrifice at 12 weeks (Figure 2C), examination of these two animals showed no hepatic tumor (last image lower panel Figure 2D).

We then studied the effect of increasing TMZ from 3 to 14 doses every 3 days alone and in combination with LB1; TMZ dosing started 10 days after LB1. The study was done in two stages: TMZ alone (n = 5) versus vehicle alone (n = 5) and TMZ plus LB1 (n = 7) versus vehicle alone (n = 7). Fourteen doses of TMZ alone delayed development and growth of the tumors from 5 to 8 weeks in all animals but tumor progression required sacrifice by week 10. LB1 plus TMZ delayed tumor growth in all animals to 12 weeks (log rank: *P*<0.0001, Figure. 2E–2F). Two of these mice required sacrifice at week 14 and 15 and 3 animals, without apparent disease as shown by MRI, (Figure 2G) were sacrificed at 16 weeks and were found to be free of hepatic tumors. Histological sections showed multiple healthy viable appearing clusters of tumor cells in livers of untreated animals but only scar tissue in damaged liver parenchyma without viable tumor cells seen in the animals having complete tumor regressions (Figure 2H).

In a third study, the effect of 14 doses of TMZ every 3 days, beginning the same day as initiation of LB1, rather than day 10 of LB1 (n = 5), was compared to a concomitant control group (n = 5). Tumor growth was suppressed or delayed in all treated animals; 2 animals required sacrificing at week 15 and 16. Three animals sacrificed at week 17 had no evidence of hepatic tumors (Figure 2I). There was no apparent drug toxicity based on observation of the mice and documented absence of significant weight loss on any treatment arm.

### Molecular and cell cycle analyses of MPC tumor cells treated with LB1, TMZ and their combination

**Figure 3:**
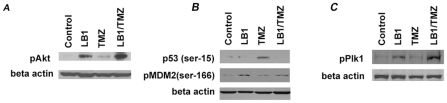
Western blot on MPC cells. Changes in pAKT, p53, pMDM2, and pPlk-1 after 24 hours treatment on MPC cells with 5 µM of LB1, 50 µM of TMZ and combination of both drugs. (A) Western blots show that LB1 exposure increases pAKT compared to control MPC cells (untreated, only vehicle). TMZ does not noticeably change the expression of pAKT and combination of LB1 and TMZ highly increases pAKT expression. (B) A demonstration of markedly increased expression of p53 after TMZ treatment but inhibition of expression by exposure to LB1. Noticeable increase in the expression of pMDM2 in MPC cells treated with LB1 alone or in combination with TMZ. (C) Noticeable increases in expression of pPlk1 in MPC cells after exposure to the combination of drugs.

We compared the extent of phosphorylation of pAkt (ser 308), polo-like kinase 1 (Plk-1), pPlk-1(thr 210), and pMDM2 (ser 166) to the relative amounts of p53 (ser 15) treated MPC cells compared to controls, and in treated compared to untreated hepatic tumors. MPC cells cultured for 24 hours and exposed to vehicle, LB1 (5 µM), TMZ (50 µM), and their combination, revealed changes in pAkt, pPlk-1, and pMDM2 and p53 expression (Figure 3A–3C). LB1 and combination of LB1 and TMZ exposure increases pAKT on MPC cells. TMZ does not noticeably change the expression of pAKT.

LB1 had no effect on expression of p53 but exposure to TMZ markedly increased expression of p53, however, LB1 alone or in combination with TMZ increased the expression of pMDM2 in MPC cells. The combination of LB1 and TMZ increased expression of pPlk-1.

**Figure 4:**
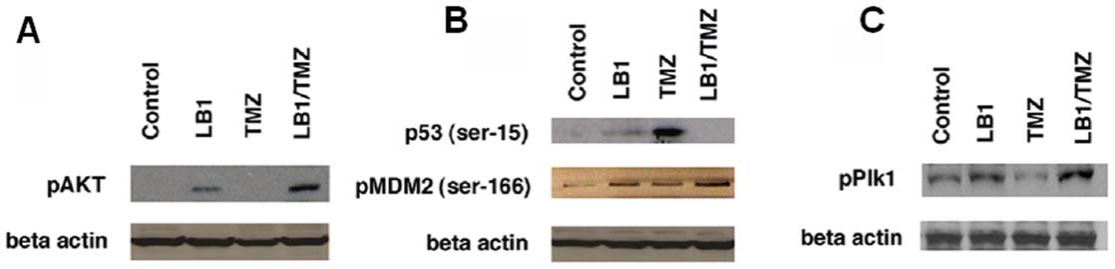
Western blot on PHEO tumors. Changes in the state of phosphorylation and abundance of small pAKT, p53, pMDM2, and pPlk-1 24 hours after treatment of mice bearing hepatic tumors with vehicle, LB1 alone at 1.5 mg/kg by gavage, TMZ alone by gavage at 80 mg/kg, of both drugs at the same doses. (A) Western blots show that LB1 exposure increases pAKT in control-untreated tumors and treated tumors. TMZ does not change the expression of pAKT and combination of LB1 plus TMZ highly increases pAKT expression. (B) Western blots demonstrate marked increased expression of p53 after TMZ but complete inhibition of this induction by exposure to LB1 accompanied by an increase in the expression of pMDM2 in tumor cells exposed to LB1 alone or in combination with TMZ (C) Expression of pPlk1 shows a marked increase in tumors after exposure to the combination of drugs.

The tumors were examined 24 hours after single administration of LB1 (1.5 mg/kg, *i.p*.), TMZ (80 mg/kg, by gavage) or combination of both drugs at the same concentrations. Comparing to GBM xenografts [21], exposure of metastatic PHEOs to LB1 alone increased pAkt, pPlk-1, and pMDM2 and had little effect on p53. Exposure to TMZ alone reduced pPlk-1 and markedly increased p53 without a change in pMDM2. The combination of LB1 and TMZ, however, prevented the TMZ-induced decrease in pPlk-1, increased the concentration of pMDM2 and eliminated p53 induction (Figure 4A–4C).

**Figure 5:**
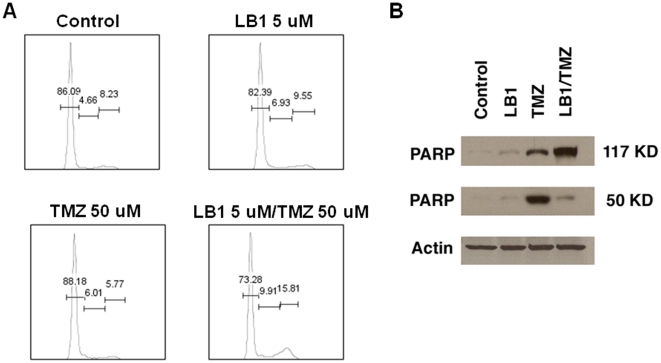
Effect of LB1 and TMZ on tumor cell cycle and apoptosis. (A) Cell cycle analysis of MPC cells in exponential growth exposed for 48 hours to vehicle alone; LB1 alone at 5 µM; TMZ at 50 µM and; LB1 at 5 µM and TMZ at 50 µM. (B) PARP expression changes in 24 hours after treatment of mice bearing hepatic tumors with vehicle, LB1 alone at 1.5 mg/kg by gavage, TMZ alone by gavage at 80 mg/kg, of both drugs at the same doses.

**Figure 6:**
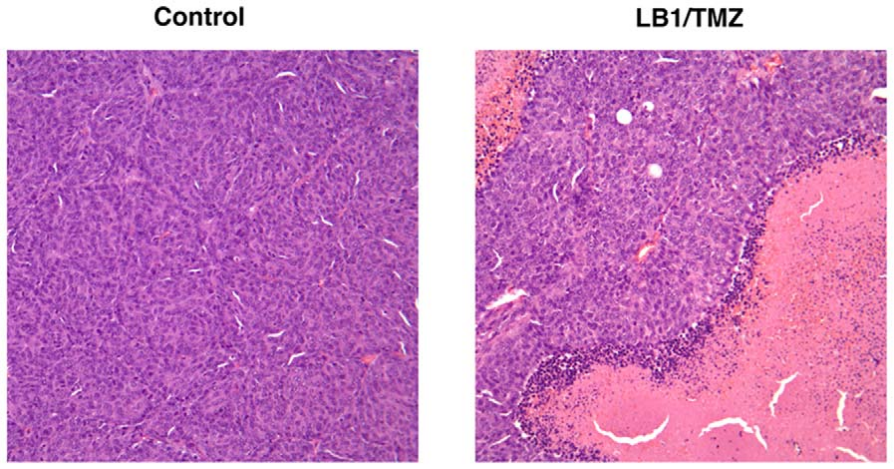
Histolopathology. Histologic features of liver PHEO lesions stained with H&E 24 hours after *i.p*. vehicle alone (left) or with *i.p.* LB1 at 1.5 mg/kg plus TMZ by gavage at 80 mg/kg (right). Exposure to a single *i.p.* injection of vehicle showed a homogeneous field of healthy appearing tumor cells, whereas combination treatment resulted in extensive necrosis of tumor cells.

We analyzed the cell cycle status of MPC cells growing exponentially in culture after exposure for 48 hours to vehicle, LB1 (5 µM), TMZ (50 µM), and combination of both drugs at the same concentrations. The majority of control cells were in G_0_/G_1_phase (86%) with less than 5% in S phase and about 8% in G_2_/M phase. Exposure to LB1 or to TMZ only modestly changed these distributions, with TMZ causing a slight increase in G_0_/G_1_ and S phase cells and a slight reduction in G_2_/M phase cells. The combination of drugs altered the cell cycle with a 15% decrease in G_0_/G_1_ and a 100% increase in cells in S and G_2_/M phases, compared to control cells (Figure 5A) indicates loss of checkpoints in G1 and G2 ordinarily induced by acute DNA-damage. Apparently, cell death proceeded by induction of mitotic catastrophe rather than by apoptosis in cells exposed to both drugs, as indicated by the absence of low molecular weight (50kD) PARP, in cells treated with both drugs as compared to TMZ alone (Figure 5B). *In vivo,* combination treatment resulted in extensive necrosis of tumor cells (Figure 6).

## Discussion

In the present study, all animals with metastatic PHEOs, treated with combination of drugs LB1 plus TMZ, had a significant delay in liver tumor growth and 40% of treated animals were found to be tumor free. There was no toxicity in mice receiving LB1 and TMZ in combination. Inhibition of PP2A was associated with prevention of G1/S phase arrest by p53 and mitotic arrest mediated by Plk-1. Simultaneous elimination of DNA damage-induced defense mechanisms, through transient pharmacologic inhibition of PP2A, is proposed as a new method for enhancing the efficacy of non-specific cancer chemotherapy regimens against a broad spectrum of low growth fraction tumors, such as PHEOs, that are commonly resistant to cytotoxic drugs.

The alterations induced by inhibition of PP2A that appeared to be responsible for changes in cell cycle parameters are mediated by increased phosphorylation of Akt-1, which in turn increases phosphorylation of pMDM2 [23], [24], and pPlk-1. Phosphorylation of MDM2 leads to degradation of p53, which, in the presence of DNA damage by a non-specific agent such as TMZ, prevents cell cycle arrest. Mitotic arrest can be caused not only by direct inhibition of microtubules during mitosis (e.g., paclitaxel-induced mitotic arrest) but also by DNA damage prior to mitosis (in the interphase of the cell cycle) [25]. Perhaps equally important is the activation, more specifically phosphorylation, of Plk-1 in the presence of DNA damage through inhibition of Plk1. Smits and colleagues showed that over-expression of activated Plk-1 can block mitotic arrest induced by DNA-damage [26]. Recently, Jang and coworkers demonstrated that a reduction in phosphorylated Plk in response to DNA damage during mitosis is mediated by PP2A and that okadaic acid, a natural product inhibitor of PP2A, inhibits the reduction of phosphorylated Plk1 induced by DNA damage [27]. We suggest that potentiating the anti-cancer activity of TMZ by inhibition of PP2A is caused by abrogation of the mitotic checkpoint by enhanced phosphorylation of Plk-1 and the simultaneous inhibition of cell cycle arrest in G1/S that results from the marked reduction in p53 driven by increased pMDM2. The net result is continued entry of cancer cells from G0/G1 into S phase and S phase into G2/M phases despite the presence of acute DNA damage that would ordinarily arrest the cell cycle in the G1 and G2 phases. It appears that this is a dynamic process by which cancer cells are prevented from completing replication over several days in the animal, perhaps accounting for the very modest effects of the drug combination in short term culture *in vitro*. Interestingly, liver tumors were growing more after 14-days of LB1 treatment even compared to control group and in several cases animals were dying faster. This observation was in correlation with cell cycle data, where tumor cells were pushed more into the S and G2/M phase. Thus, LB1 sensitized more tumor cells to the same cell cycle stage where targeted treatment with TMZ would be more effective. TMZ alone did not prevent from tumors' development, however, combined with LB1 was survival of animals significant. These results also confirm cell cycle data, where drug combination altered the cell cycle with a 15% decrease in G0/G1 phase, and 100% increase in S and G2/M phase.

We presume that the cancer cell genome has several, if not multiple acquired defects in genes for proteins controlling cell cycle, rendering cancer cells less tolerant than normal cells to modulation of phosphorylation mediated through signal transduction [21], [28]. Goodzari et al. presented that global perturbations in regulatory elements of human cancer, showed a general over-expression of mitotic pathways, including 11 associated with DNA repair in cancer cells compared to normal cells [29].

Toxicity permitting, the combination of a small molecule inhibitor of PP2A and an agent targeting DNA, such as chemotherapy and x-radiation, may provide newer and more effective treatments for a broad spectrum of human cancers despite large differences in their degrees of differentiation and growth rates. The proposed cancer therapy approach in the present study is novel and has far reaching implications as a major advancement in the treatment of metastatic cancer such as metastatic PHEO and PGL. Our results indicate that LB1 helps to potentiate the cytotoxicity effect of chemotherapy against metastatic PHEO in a mouse model. This finding can be extended to the clinic in the treatment of resistant PHEO and PGL as there is currently no successful treatment of this disease.

## Methods

### Materials and culture

Mouse PHEO (MPC 4/30PRR) cells were a gift from A.S. Tischler [22]. MPC cells were maintained in Dulbecco's Modified Minimum Essential Medium (DMEM, Gibco, USA, heat-inactivated horse serum (Hyclone Logan, UT), fetal bovine serum (GIBCO, Grand Island, NY), penicillin (10,000 units/ml)/streptomycin (10,000 units/ml) (GIBCO, Grand Island, NY), and maintained at 37°C with 5% CO_2_. MPC cells were grown in tissue culture dishes without collagen. We have verified that MPC cells are uninfected with mycoplasma. Prior to injection into mice, MPC were incubated in 0.05% trypsin (GIBCO), and gently rocked at 37°C until they detached from the flask. Cells were transferred to a 15 ml conical tube containing fresh medium and then washed and re-suspended in 100 µl of medium before injection into nude mice. TMZ was purchased from Sigma-Aldrich Co., St. Louis, MO. LB1 (also designated LB-100 [30]) was provided by Lixte Biotechnology Holdings, Inc. (LBHI).

### Cytotoxicity assay

The antiproliferative activity of LB1 and TMZ on cells at varying concentrations was examined by the XTT assay (Cell Proliferation Kit II, Roche Diagnostics Corporation, Roche Applied Science, Indianapolis, IN). After dissociation, MPC cells (passage 25–28, *in vivo* and *in vitro*) were seeded in 96-well plates coated with collagen (15,000 cells/well) and incubated at 37°C. LB1 in concentrations of 2, 5, 10, 15 and 20 µM and TMZ in concentrations of 100, 200, 300, 400, 500 and 600 µM and in various combinations were added 24 hours after cell seeding and incubated at 37°C. To test a potential interference of XTT kit reagents and drugs, LB1 and TMZ were tested in absence of cells and evaluated with XTT reagent kit. Antiproliferative activity was measured 24, 48 and 72 hours after drug administration. The XTT labeling mixture was added, and the plates were incubated for an additional 6–24 hours, after which spectrophotometric absorbance was measured with a microplate reader (Bio-Rad Laboratories, Philadelphia, PA) according to the manufacturer's instructions. All experiments were performed in triplicate and repeated at least twice. The concentration of drugs that reduced cell survival by 50% (IC50) as compared to controls was calculated. The survival of treated cells was expressed as a percentage of control (vehicle treated) cultures.

### Synergy analysis of combined drug effects

Drug synergy was determined from median effect analysis by Chou-Talalay equations [31] using the CalcuSyn software (Biosoft). Using cell proliferation assay and computerized data, Combination index (CI) was generated between LB1 and TMZ drugs. Additivity was then defined as CI = 1; synergy as CI<1; and antagonism as CI>1.

### *In vivo* studies

All animal studies were conducted in accordance with the principles and procedures outlined in the National Institute of Health Guide for the Care and Use of Animals, and approved by the Eunice Kennedy Shriver National Institute of Child Health and Human Development Animal Care and Use Committee. Female athymic nude mice (NCr-nu) were obtained from Taconic Inc. (Germantown, MD) and were housed in a pathogen-free facility. The mice were acclimated for at least 3 days in the animal facility in which the appropriate temperature, humidity and light cycle (6:00 A.M. – 6:00 P.M) were controlled, with *ad libitum* access to food and water. Each experimental group consisted of six to ten-week old mice. Non-anaesthetized mice were intravenously injected in the tail vein using a 1 ml syringe and 30 1/2 gauge needles with 1×10^6^ MPC cells (passage 25–28) in 100 µl of medium. Mice were monitored daily and examined by weekly MRI for the number and extent of tumor growth in the liver.

### Drug administration

LB1 and vehicle were given via a mini-osmotic pump (model 1002 Alzet, Cupertino) that was implanted intraperitoneally while the animals were under anesthesia. The pump was filled under sterile conditions, per the manufacturer's instructions, with either 100 µl of LB1 (0.03 mg/day dissolved in PBS) or PBS as vehicle into control mice. The pumps had a mean flow rate of 0.25 µl/hour for the 2-week study duration. TMZ (80 mg/kg) was given by gavage every 3 days at either 3 or 14 doses. Animals were weighed once a week and monitored daily for possible side effects of the drugs. Those with significant tumor burden were sacrificed.

It is important to note that the metastatic PHEO model presents practical challenges since the stress imposed by manipulation cause the release of catecholamines from tumors and frequent animal demise [32]. Therefore, we have decided to use the mini-osmotic pump for LB1 administration in order to minimize animal handling. This approach was also based on our previous experience using osmotic pumps [33], when excellent results were obtained delivering drugs systematically. In the same manner, frequent *i.p.* injections would be difficult to perform due to possible tumor perforation and hemorrhage (liver tumors filled the entire peritoneal cavity).

### Magnetic resonance imaging

Tumor growth in the liver was monitored by MRI once a week starting at the 3^rd^ week after the MPC cells *i.v* injection. Usually, tumors started to be visible in week 4 and fully measurable in week 5. For MRI studies, mice were anesthetized with inhaled isoflurane/O_2_ at a dose of 1.5–5% v/v adjusted to produce a respiratory rate of approximately 40 breaths per minute. Mice were placed in the prone position and kept warm during the scanning as well as during post-scan recovery from anesthesia. Fat-suppressed, T_2_-weighted spin echo images were acquired on a clinical Philips Intera 3.0 Telsa (3T) system, using a dedicated 40 mm inner diameter solenoid coil (Philips, Best, Netherlands). A total of 40 slices were acquired with TE/TR 65/4500 ms; slice thickness was 0.5 mm, 0.16×0.16 mm^2^ in-plane resolutions with a scan time of 5–7 minutes for two signal averages. MRI data was acquired and reconstructed using the scanner software (Intera, Philips Medical System, Best, Netherlands). Quantitative measurements of volume changes were obtained using the ImageJ and MIPAV software [34]. The size and volume measurements of liver tumors were performed as described before [35] Measurements were taken from groups containing five animals.

### Histological, biochemical and molecular characterization

Animals were euthanized using CO_2_ inhalation and cervical dislocation. Tumors were resected from the excised liver and then formalin-fixed, paraffin-embedded and sectioned (10 µm). Hematoxylin and eosin (H&E) staining was performed.

### Western blot studies

Primary antibodies to p-Akt (Thr308), p53 (ser-15), PARP and MDM2 (ser-166) were purchased from Cell Signaling Technology Inc. (Danvers, MA). Plk1 (T210) was purchased from Abcam Inc. (Cambridge, MA). To determine the effects of LB1 on Akt, Plk1, p53, and MDM2, cultured MPC cells were treated with vehicle control, 5 µM LB1, 50 µM of TMZ and their combination. After 24 hours of treatment, cultured cells were harvested and resuspended in T-PER solution, sonicated, and centrifuged. Animals bearing hepatic tumors were treated with vehicle, LB1 alone, TMZ alone and the combination of LB1 and TMZ. After 24 hours, animals were sacrificed and tumor tissue was sectioned, resuspended in T-PER solution, sonicated and centrifuged. The protein concentration in each sample was measured by a colorimetric assay (Bio-Rad Protein Assay Kit) (Bio-Rad; Hercules, CA). Expression of specific proteins in each sample was determined via Western blotting using primary antibodies. Detection of protein-bound primary antibodies was performed with a horseradish peroxidase-conjugated secondary antibody specific to rabbit immunoglobulin and an enhanced chemiluminescence system.

### Cell cycle analyses

MPC cultured cells were treated with vehicle or the test regimen for 48 hours. Following culture, cells were fixed with 70 ethanol overnight at −20°C. The fixed cells were stained with 10 µg/ml PI and 1 µg/ml RNase for 30 minutes and analyzed by FACS [36].

### Statistical analysis

Results from the XTT cell viability assay and volume measurements of liver lesions are presented as mean±standard error of the mean (SEM). Statistical differences between groups were assessed by ANOVA, followed by Student-Neuman-Keuls test for group comparison. The level of statistical significance was set at *P*<0.05. Differences in animal survival rates were determined by Kaplan-Meier Analysis. A probability value of *P*<0.05 was considered significant.
